# C1s targeting antibodies inhibit the growth of cutaneous squamous carcinoma cells

**DOI:** 10.1038/s41598-024-64088-3

**Published:** 2024-06-12

**Authors:** Liisa Nissinen, Pilvi Riihilä, Kristina Viiklepp, Vaishnavi Rajagopal, Michael J. Storek, Veli-Matti Kähäri

**Affiliations:** 1 Department of Dermatology and FICAN West Cancer Centre Research Laboratory, University of Turku and Turku University Hospital, Hämeentie 11 TE6, 20520 Turku, Finland; 2 Sanofi, Cambridge, MA USA

**Keywords:** Squamous cell carcinoma, Complement, C1s, Targeting antibodies, Therapeutic target, Squamous cell carcinoma, Drug development

## Abstract

Cutaneous squamous cell carcinoma (cSCC) is the most common metastatic skin cancer. The incidence of cSCC is increasing globally and the prognosis of metastatic disease is poor. Currently there are no specific targeted therapies for advanced or metastatic cSCC. We have previously shown abundant expression of the complement classical pathway C1 complex components, serine proteases C1r and C1s in tumor cells in invasive cSCCs in vivo, whereas the expression of C1r and C1s was lower in cSCCs in situ, actinic keratoses and in normal skin. We have also shown that knockdown of C1s expression results in decreased viability and growth of cSCC cells by promoting apoptosis both in culture and in vivo. Here, we have studied the effect of specific IgG2a mouse monoclonal antibodies TNT003 and TNT005 targeting human C1s in five primary non-metastatic and three metastatic cSCC cell lines that show intracellular expression of C1s and secretion of C1s into the cell culture media. Treatment of cSCC cells with TNT003 and TNT005 significantly inhibited their growth and viability and promoted apoptosis of cSCC cells. These data indicate that TNT003 and TNT005 inhibit cSCC cell growth in culture and warrant further investigation of C1s targeted inhibition in additional in vitro and in vivo models of cSCC.

## Introduction

Cutaneous squamous cell carcinoma (cSCC), which originates from epidermal keratinocytes, is the most common metastatic skin cancer and its incidence is increasing worldwide^1^. The estimated metastasis rate of cSCC is 3–5%, and the prognosis of patients with metastatic disease is poor, as over 70% of patients with metastatic cSCC will die within 3 years^2,3^. Furthermore, cSCC has been estimated to cause 20% of skin cancer‒related mortality^1^. A UV-induced premalignant lesion, actinic keratosis may develop to malignant cSCC in situ (Bowen’s disease) and finally to invasive and metastatic cSCC^4^. The major risk factor for cSCC is long-term exposure to UV-radiation. Other risk factors for cSCC development and progression are chronic inflammation, chronic dermal ulcers, and immunosuppression^4^. The complement system is a part of both innate and acquired immune systems^5,6^. The complement system may be activated through classical, lectin, or alternative pathways and all of these pathways converge into cleavage of C3 and activation of terminal pathway and formation of lytic membrane attack complex^7^. C1s is a part of classical pathway initiating C1qr_2_s_2_ complex. Binding of C1q to target molecule induces autocatalytic activation of the serine proteinase C1r, which in turn activates another serine proteinase C1s^8^. There is also increasing evidence that components of C1 complex exert cancer-promoting functions and play a role in cancer progression independently of the complement system activation in a non-canonical manner^7,9,10^. C1q has been noted to be expressed in the microenvironment of several human malignant tumors and to contribute to tumor progression without activation of complement classical pathway^11^.

We have previously shown marked upregulation of C1s expression in tumor cells in cSCC in vivo and in cSCC cell lines in culture^12^. Knockdown of C1s expression resulted in decreased viability and growth of cSCC cells by promoting apoptosis both in culture and in vivo. C2 or C4 was not present in cSCC cultures indicating non-canonical function of C1s in cSCC cells^13^.

In this study, we have investigated the effect of C1s targeted antibodies TNT003 and TNT005 on cSCC cell growth and viability. Treatment of cSCC cells with TNT003 or TNT005 significantly inhibited their growth and promoted apoptosis. These data demonstrate that TNT003 and TNT005 are potent inhibitors of cSCC cell growth in culture and provide the rationale for additional studies in relevant in vitro and in vivo disease models to support the development of C1s targeted inhibitors for the treatment of locally advanced and metastatic cSCCs.

## Materials and methods

### Ethical issues

The use of tumor derived SCC cell lines was approved by the Ethics Committee of the Hospital District of Southwest Finland (187/2006). All participants gave their written informed consent, and the study was performed with the permission of Turku University Hospital, according to the Declaration of Helsinki.

### C1s targeting antibodies TNT003 and TNT005

TNT003 and TNT005 were provided by Sanofi. Both antibodies inhibit the classical complement pathway. TNT003 binds to both the active and inactive forms of C1s. In contrast, TNT005 is highly specific for the active form. They are both murine IgG2a antibodies identified using the traditional hybridoma technology, as previously described^14,15^. Briefly, mice were immunized with human active C1s protein and a hybridoma library was generated and screened. Antibodies were purified from hybridoma supernatants using Protein A chromatography. The control antibody IgG 068 (IgG) is a mutated version of TNT003 that lacks target binding.

### Cell cultures

Primary non-metastatic (UT-SCC-12A, UT-SCC-91, UT-SCC-105, UT-SCC-111 and UT-SCC-118) and metastatic (UT-SCC-7, UT-SCC-59A and UT-SCC-115) cSCC cell lines were established from surgically removed cSCCs in Turku University Hospital^12,16,17^ (Supplementary Table S1). cSCC cells were cultured as previously described^12^. The authenticity of all cSCC cell lines has been verified by short tandem repeat profiling^16^. The immortalized non-tumorigenic human keratinocyte–derived cell line (HaCaT) was kindly provided by Dr Norbert Fusenig (Deutsche Krebsforschungszentrum, Heidelberg, Germany). HaCaT cells were cultured as previously described^13^. For antibody treatment, indicated concentrations of control IgG and C1s targeting antibodies TNT003 and TNT005 were added to cSCCs cell cultures in serum free conditions for different periods of time.

### Western blotting

For western blot analysis, samples were fractionated in 10% SDS–polyacrylamide gel and transferred onto nitrocellulose membrane (Amersham Biosciences, Piscataway, NJ, USA), as previously described^12^. Production of C1r and C1s by cSCC cells was determined by western blotting analysis of conditioned media or total cell lysates using specific polyclonal rabbit anti-C1s (HPA018852; Sigma-Aldrich) and anti-C1r (HPA001551; Sigma-Aldrich) antibodies. Cell lysates were also analyzed with antibodies specific for phosphorylated protein kinase B (Akt), phosphorylated extracellular signal–regulated kinase (ERK)-1/2, total ERK1/2 (9271S, 9101, and 9102, respectively; all from Cell Signaling Technology, Beverly, MA), and total Akt (sc-1618, Santa Cruz Biotechnology, Santa Cruz, CA). TIMP-1 (Ab-1; Merck Millipore) or β-actin (A1978; Sigma-Aldrich) antibody was used to determine protein loading.

### Cell growth assay

Cells were seeded (7.5 × 10^3^ cells/well) on 96-well plates. The IncuCyte S3 real-time cell imaging system (Essen BioScience, Ann Arbor, MI) was used to study the growth of cSCC cells. Images were taken every 2 h by IncuCyte S3, and the relative confluence was analyzed by the instrument. Experiments were carried out with 5–8 parallel wells at every time point with six cSCC cell lines (UT-SCC-59A, -115, -12A, -105, -118 and -7).

### Cell viability assay

Cutaneous SCC (1.0 × 10^4^ cells/well) or HaCaT (7.5 × 10^3^ cells/well) cells were seeded on 96-well plates. The number of viable cells was determined using a Cell Counting Kit-8 (CCK-8, Dojindo, Japan). The experiments were carried out with 6–8 parallel wells in every time point with six cSCC cell lines (UT-SCC-59A, -115, -12A, -105, -118 and -7) and non-tumorigenic human keratinocyte–derived HaCaT cells.

### Apoptosis assays

Apoptotic cells were detected after 24 h treatment with antibodies by using In Situ Cell Death Detection Kit (Roche). Number of TUNEL positive cells was counted from 4 parallel image fields / cell line using 20 × objective and compared to total cell number visualized by Hoechst 33342 (Invitrogen). The experiments were carried out with seven cSCC cell lines (UT-SCC-59A, -115, -12A, -91, -105, -118 and -7).

## Results

### C1s and C1r expression by cSCC cell lines

**Figure 1:**
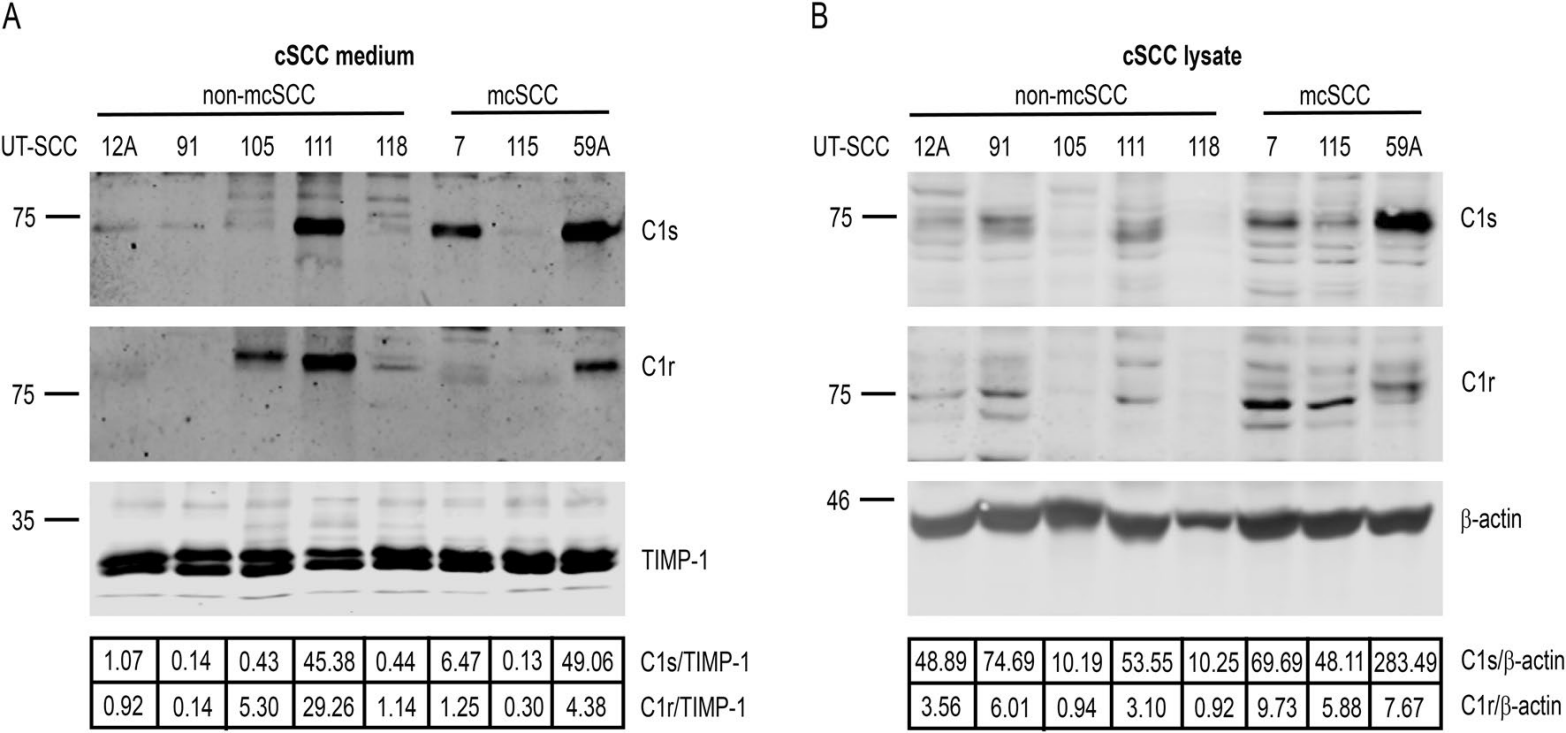
C1s and C1r expression in cutaneous squamous cell carcinoma (cSCC) cells. **A** Conditioned media and **B** total cell lysates of non-metastatic (non-mcSCC) and metastatic (mcSCC) cSCC cell lines were collected from cell growth experiments and analyzed by western blotting. The levels of C1s and C1r are shown. TIMP-1 (**A**) and β-actin (**B**) were determined as the loading controls. Quantitations of the western blots corrected for loading controls are shown below the panels.

The production of C1s and C1r by cSCC cell lines was determined by western blotting of conditioned media (Fig. 1A) and cell lysates (Fig. 1B). The secretion of C1s to cell culture media was noted in all cSCC cell lines examined (Fig. 1A). Most prominent secretion of C1s was detected in two metastatic cSCC cell lines UT-SCC-7 and -59A and in a primary non-metastatic cSCC cell line UT-SCC-111 (Fig. 1A). The levels of C1s were also detected in whole cell lysates (Fig. 1B) and in UT-SCC-115 and UT-SCC-91 the intracellular levels of C1s were higher than the secreted levels of C1s compared to other cSCC cell lines (Fig. 1A,B). C1r was secreted to the medium or noted as intracellularly in the majority of cSCC cell lines indicating the possibility to activate C1s (Fig. 1).

### C1s targeted antibodies TNT003 and TNT005 inhibit growth and viability of cSCC cells

**Figure 2:**
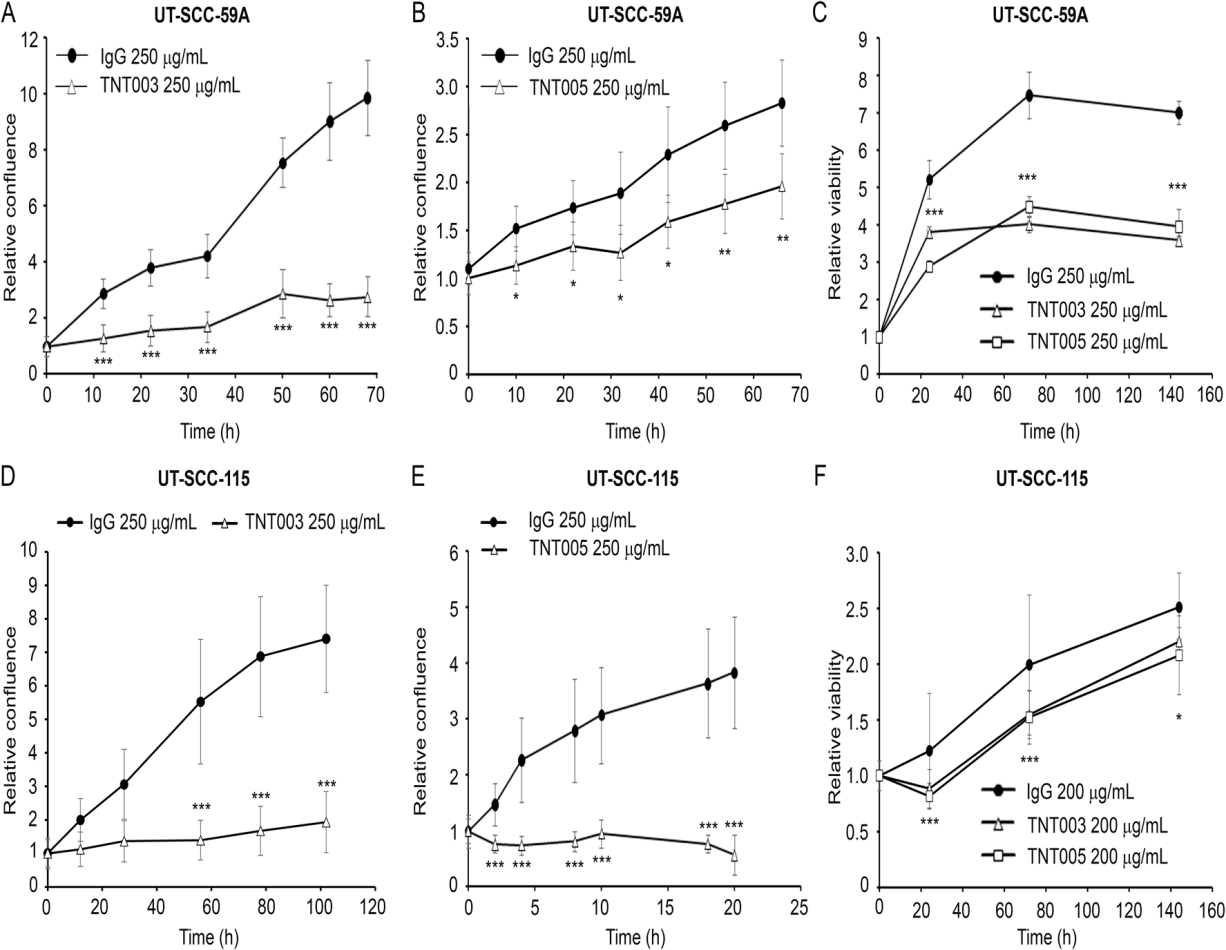
C1s targeted antibodies TNT003 and TNT005 inhibit the growth and viability of cutaneous squamous cell carcinoma (cSCC) cells. **A**, **B** Metastatic cSCC cell lines UT-SCC-59A and **D**, **E** UT-SCC-115 (7.5 × 10^3^ cells/well) were plated on 96-well plates. Control antibody (IgG) and C1s targeting antibodies TNT003 and TNT005 were added to cells under serum free conditions. The IncuCyte S3 real-time cell imaging system was used to study the growth of cSCC cells and the relative confluence was analyzed by the instrument (n = 6–8). **C** UT-SCC-59A and **F** UT-SCC-115 cells (1.0 × 10^4^ cells/well) were plated on 96-well plates. Control antibody (IgG) and C1s targeting antibodies TNT003 and TNT005 were added to cells in serum free conditions. The number of cSCC cells was determined at time points indicated using CCK-8 assay (n = 6–8). *p < 0.05, **p < 0.01, ***p < 0.001, Student’s t-test.

C1s was targeted with antibodies TNT003 and TNT005 to investigate the effect of C1s in cSCC cell growth and viability. The dose response of the effect of the antibodies on cSCC cell viability was investigated (Supplementary Fig. S1) and the maximum concentration 250 µg/mL was selected based on previous publications with other cell lines^18^. Treatment of metastatic cSCC cell lines UT-SCC-59A and UT-SCC-115 with TNT003 and TNT005 (250 µg/mL) significantly inhibited the growth (Fig. 2A,B,D,E) and viability (Fig. 2C,F) of cSCC cells compared to control antibody (IgG). TNT003 and TNT005 (250 µg/mL) also inhibited the growth of primary UT-SCC-105, UT-SCC-12A, UT-SCC-118 and metastatic UT-SCC-7 cell lines (Supplementary Fig. S2). Additionally, TNT003 inhibited the growth of UT-SCC-115, UT-SCC-118 and UT-SCC-7 already at 200 µg/mL concentration (Supplementary Fig. S2A,D,E). Furthermore, inhibition of the growth of UT-SCC-7 cell line with TNT005 was detected already at 200 µg/mL concentration (Supplementary Fig. S2E). TNT003 and TNT005 (200µg/mL) inhibited the viability of UT-SCC-12A, UT-SCC-118, UT-SCC-105 and UT-SCC-7 cell lines (Supplementary Fig. S3).

The effect of TNT003 and TNT005 on the expression of C1s and C1r was investigated by western blotting of lysates of cSCC cells treated with the antibodies for 72 h (Supplementary Fig. S4). The expression of C1s and C1r was not markedly regulated by C1s targeted antibodies TNT003 or TNT005 in cSCC cells (Supplementary Fig. S4).

Immortalized non-tumorigenic keratinocyte–derived cell line (HaCaT) was utilized to determine the effect of TNT003 and TNT005 (250 µg/mL) on viability of non-malignant keratinocyte-derived cells. The production of C1s and C1r by HaCaT and UT-SCC-59A cells was examined by western blotting of conditioned media (Supplementary Fig. S5A) and cell lysates (Supplementary Fig. S5B). No expression was noted in HaCaT cells, whereas the cSCC cell line UT-SCC-59A used as positive control showed expression of both C1s and C1r (Supplementary Fig. S5A,B). Treatment with TNT003 or TNT005 had no effect on the viability of HaCaT cells (Supplementary Fig. S5C), whereas treatment of UT-SCC-59A cells in parallel with TNT003 and TNT005 (250 µg/mL) significantly inhibited their viability (Supplementary Fig. S5D).

### C1s targeted antibodies TNT003 and TNT005 promote apoptosis of cSCC cells

**Figure 3:**
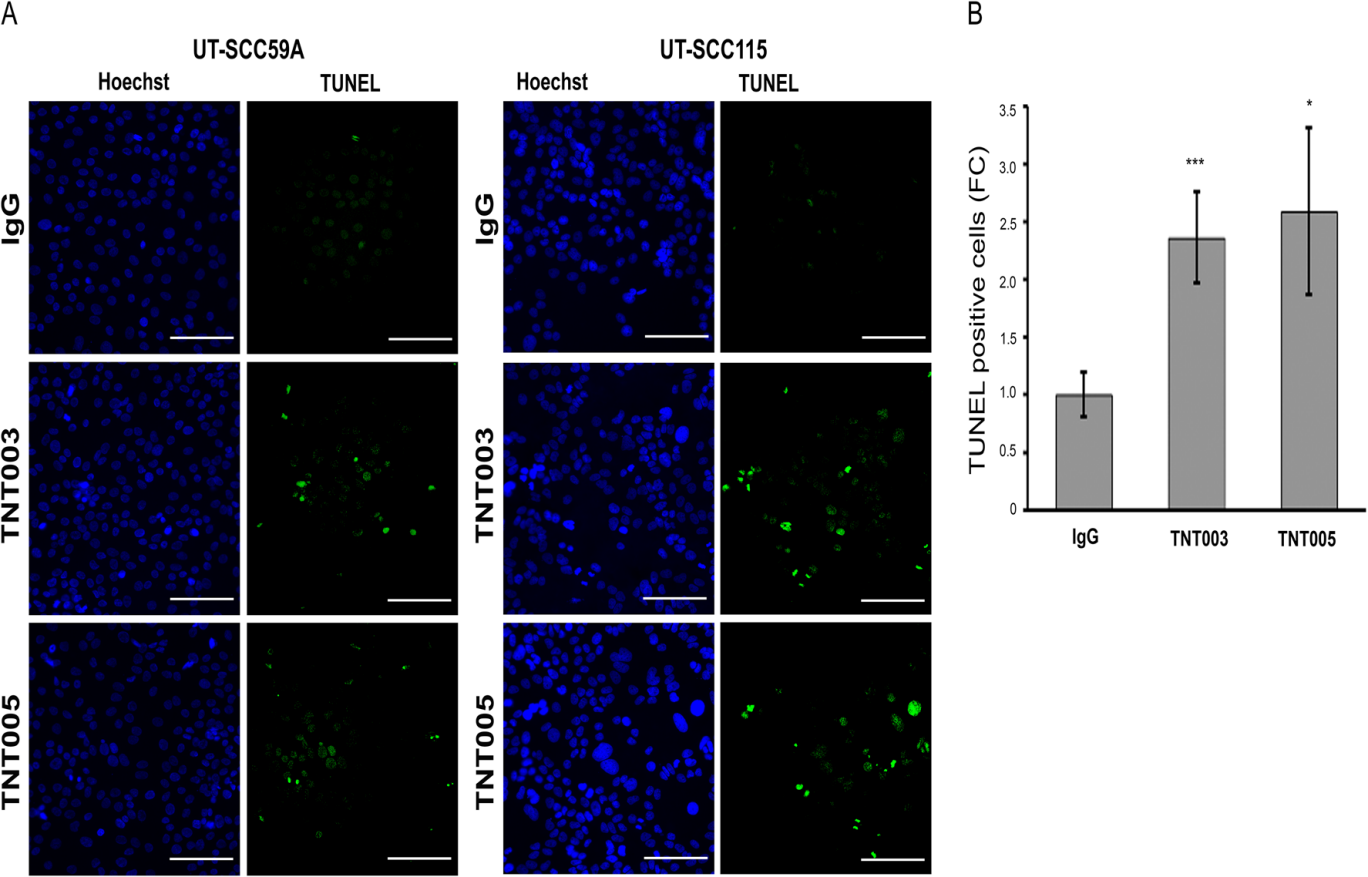
C1s targeted antibodies TNT003 and TNT005 promote apoptosis of cutaneous squamous cell carcinoma (cSCC) cells. **A** Representative images of Hoechst and TUNEL stainings for metastatic cSCC cell lines UT-SCC-59A and UT-SCC-115 are shown. Scale bar 100 µm. **B** Seven cSCC cell lines (UT-SCC-59A, -115, -12A, -91, -105, -118 and -7) were treated with control antibody (IgG) and C1s targeting antibodies TNT003 and TNT005 (250 µg/mL) for 24 h under serum free conditions, apoptotic cells were detected with TUNEL staining, and the relative number of TUNEL‐positive cells was counted (n = 7). *p < 0.05, ***p < 0.001, Student's t-test.

Knockdown of C1s has been shown to promote apoptosis of cSCC cells in culture and in vivo^12^. Therfore, we investigated, whether TNT003 and TNT005 could induce apoptosis of cSCC cells. The representative images of these experiments with cSCC cell lines are shown in Fig. 3A and Supplementary Fig S6. Increased number of TUNEL‐positive apoptotic cSCC cells was detected after 24 h treatment with 250 µg/mL C1s targeting antibodies TNT003 and TNT005 compared to control antibody (IgG) (Fig. 3B).

### C1s targeted antibodies TNT003 and TNT005 inhibit ERK1/2 and Akt signaling pathways in cSCC cells

**Figure 4:**
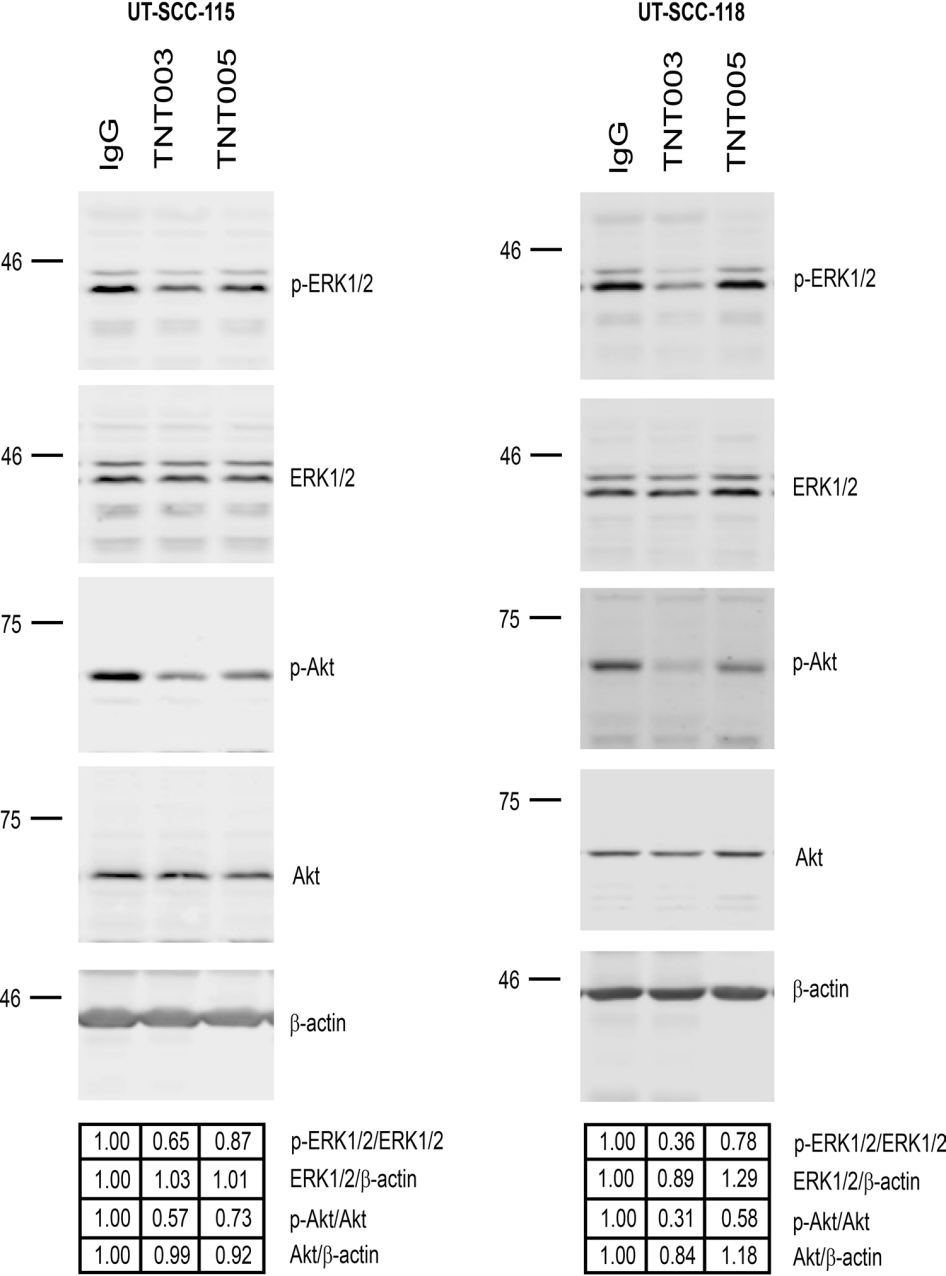
C1s targeted antibodies TNT003 and TNT005 inhibit ERK1/2 and Akt signaling pathways in cutaneous squamous cell carcinoma (cSCC) cells. **A** Metastatic cSCC cells (UT-SCC-115) and **B** non-metastatic (UT-SCC-118) cells were treated with control antibody (IgG) and C1s targeting antibodies TNT003 and TNT005 (250 µg/mL) in serum free conditions and the levels of phosphorylated Akt (p‐Akt), phosphorylated extracellular signal‐related kinase 1/2 (p‐ERK1/2), total Akt, total ERK1/2 and IgG were analyzed in cell lysates by western blotting at time points indicated. Quantitations of the western blots corrected for loading controls are shown below the panels.

Previously, C1s knockdown with specific siRNAs has been demonstrated to decrease the phosphorylation of cSCC cell growth and viability related signaling pathway molecules ERK1/2 and Akt^12^. Therefore, we studied the effect of TNT003 and TNT005 on these signaling pathways. Analysis of cell lysates by western blotting after 48 h treatment of UT-SCC-115 (Fig. 4A) and UT-SCC-118 (Fig. 4B) cell cultures with 250 µg/mL control antibody (IgG), TNT003 and TNT005 showed that the levels of phosphorylated Akt and phosphorylated ERK1/2 were decreased, indicating that C1s targeting antibodies inhibit the activation of the PI3K and ERK1/2 signaling pathways. Interestingly, IgG was detected in cell lysates of cSCC cells treated with control IgG, TNT003 and TNT005 indicating internalization of these antibodies by cSCC cells (Fig. 4).

## Discussion

Complement system is an important part of innate immunity. Complement molecules have been shown to play a major role in tumor microenvironment, but also intracellular functions for complement have been discovered^10,19^. Recent studies emphasize the non-canonical, cascade-unrelated, role of complement in cancer^10,19–23^. In cSCC, upregulated expression of several complement molecules by tumor cells has been demonstrated^7^. Tumor cell‒derived complement classical pathway C1 complex components, C1r and C1s and alternative pathway components C3, FB and FD and complement inhibitors FH and FI have been revealed to regulate the progression of cSCC at least partly in non-canonical manner^12,13,24–26^. In complement-targeted therapies the major focus has been in inflammatory disorders and several complement pathway molecule and inhibitor targeted compounds are in clinical trials^7^. They might offer therapeutic approaches also to cancer.

Previous studies show that C1s is abundantly expressed in tumor cells in invasive cSCCs in vivo, whereas the expression of C1r and C1s is lower in cSCC in situ, actinic keratosis and normal skin^12^. In this study we took a step forward to investigate the possibility to target human C1s on cSCC cells in culture. We first detected secreted C1s and C1r in cell culture media of cSCC cells, as shown previously^12^. There is also increasing evidence that complement molecules may function in an intracellular, non-canonical manner. The cleavage of intracellular C3 has been demonstrated in several studies and the biological relevance of the cleavage product C3a in cellular function is undeniable, but the mechanism is still poorly understood^27^. Intracellular complement inhibitor FH has been detected to promote tumor cell proliferation and migration in cancer cells, such as lung adenocarcinoma^28^. Additionally, intracellular C1s has been noted to modify the tumor cell phenotype e.g. in clear cell renal cell carcinoma^29^. Thus, we wanted to study the levels of C1s and C1r in cSCC cell lysates. We noted the expression of C1s in cSCC cell lines and in some cSCC cell lines intracellular levels of C1s were higher than the secreted C1s levels. Additionally, C1r was secreted to the medium or noted as intracellular in the cSCC cell lines that also express C1s. This indicates that C1s may be activated by C1r in these cells, although no correlation between C1r and C1s protein levels produced by cSCC cells has been noted in previous study^12^. Our data indicate that C1s might also function intracellularly by non-canonical manner in cSCC cells in culture. The fact that no C1q nor downstream complement classical pathway components C2 or C4 are present in cell culture conditions also emphasizes a non-canonical function of C1s in cSCC cells^12^. Interestingly, the C1s targeted antibodies were detected in cell lysates indicating internalization of the antibodies and potential inhibition of intracellular C1s.

There are several therapeutic strategies to target complement system compounds and inhibitors, such as small molecule inhibitors (SMIs), peptides, and monoclonal antibodies. They are at different stages of drug development both in clinical trials and in the preclinical phase^7,30^. We have shown that a SMI targeted against FD potently inhibits growth of cSCC cells^26^. C1q binding peptides to block C1r and C1s activation have been developed but selective inhibition of serine proteinases C1s and C1r with SMIs has been challenging^30–32^. The development of small molecular inhibitors against C1s is underway with the help of machine learning-based virtual screening and with combination of in silico and in vitro approaches^33,34^. However, until now antibodies, either monoclonal or engineered, have been the most advanced agents to block complement activity^30^.

In this study we investigated the possibility to target human C1s on cSCC cells by specific IgG2a mouse monoclonal antibodies TNT003 and TNT005. These antibodies have been shown to be effective and safe inhibitors of complement classical pathway in vivo and an inhibitory effect of the humanized versions of these antibodies, sutimlimab and riliprubart, respectively, on classical pathway activation has also been demonstrated in clinical trials^15,35–37^. Sutimlimab has been approved by FDA and EMA for treatment of cold agglutinin disease (CAD)^30^. Riliprubart is currently being tested in several phase 3 clinical trials in CAD, chronic inflammatory demyelinating polyneuropathy (CIDP) and antibody-mediated rejection globally with no major adverse events identified^38^. However, systemic inhibition of C1s in the context of cSCC remains to be tested. Here, we investigated the effect of C1s targeting antibodies on cSCC cells that show secretion of C1s into cell culture medium and intracellular expression of C1s. The results reveal that TNT003 and TNT005 potentially inhibit the growth and viability of cSCC cells regardless of whether C1s expression is high in medium or cell lysate. The maximum effect on cSCC cell viability was reached at 250 µg/mL concentration. Our previous findings demonstrated, that knockdown of C1s decreased growth and viability of cSCC cells in culture and in xenograft model in vivo^12^. Knockdown of C1s has also been shown to promote apoptosis of cSCC cells^12^. In this respect, we also investigated whether TNT003 and TNT005 could induce apoptosis of cSCC cells. We found out that treatment of cSCC cells with TNT003 or TNT005 promoted the apoptosis of these cells indicating that inhibition of C1s with targeted antibodies has similar effect on cSCC cells as knockdown of C1s expression by specific siRNAs. We also examined the effect of the antibodies on the expression of C1s and C1r in cSCC cells and noted that the antibodies had no marked effect on the expression of C1s and C1r indicating that TNT003 and TNT005 regulate only the function of C1s and not the expression levels of this molecule.

To study the specificity of C1s targeted antibodies an immortalized non-tumorigenic keratinocyte–derived HaCaT cell line that does not express C1s or C1r was utilized to determine the effect of TNT003 and TNT005 on viability of keratinocyte-derived cells. TNT003 and TNT005 were shown to be specific for cSCC cell lines since no inhibition on viability of HaCaT cells was detected. This is in accordance with previous results showing, that C1s siRNA treatment of normal human epidermal keratinocytes had no effect on the growth of keratinocytes^12^.

Previously C1s knockdown with siRNA has been demonstrated to regulate the genes involved with gene ontology terms related to signaling pathways regulating MAP kinase activity and phosphatidylinositol‐4,5‐bisphosphate 3‐kinase activity^12^. Therefore, we studied the effect of TNT003 and TNT005 on these signaling pathways. Using western blotting, we detected a decrease in ERK1/2 and Akt phosphorylation after treatment of UT-SCC-115 and UT-SCC-118 cells with C1s targeted antibodies. Although the concentrations required for the response with TNT003 and TNT005 were high (≥ 200 µg/mL) in cSCC cell culture conditions, these results indicate that it may be possible to target C1s in advanced and metastatic cSCC. Developing small molecule inhibitors against C1s might be one strategy to inhibit its function.

In summary, in this study we show in vitro proof of principle that C1s inhibition by monoclonal antibodies TNT003 and TNT005 decreases the growth and viability of cSCC cells by inducing apoptosis. We also reveal that in addition to secretion of C1s and C1r by cSCC cells there is also intracellular expression of C1s and C1r. Furthermore, the antibodies are present in lysates of treated cSCC cells and thus they might target intracellular C1s. These findings warrant further studies on the feasibility of targeting C1s in the treatment of unresectable and metastatic cSCCs.

### Supplementary Information

Supplementary Information.

## Data Availability

In response to reasonable requests, noncommercially available materials that Sanofi has the right to provide, will be made available to not-for-profit or academic requesters upon completion of a material transfer agreement. Requests may be made by contacting Dr. Michael Storek at Michael.Storek@Sanofi.com.

## Abbreviations

CAD: Cold agglutinin disease
CIDP: Chronic inflammatory demyelinating polyneuropathy
cSCC: Cutaneous squamous cell carcinoma
C1s: Complement component 1s
C1r: Complement component 1r
C3: Complement component 3
FB: Factor B
FD: Factor D
FI: Factor I
FH: Factor H
SMI: Small molecule inhibitor
