# Supplementary material for: C1s targeting antibodies inhibit the growth of cutaneous squamous carcinoma cells
Source: Sci Rep. 2024 Jun 12;14:13465. doi: 10.1038/s41598-024-64088-3 (PMC11169539; doi:10.1038/s41598-024-64088-3)
Supplement: Supplementary file 1 — Supplementary Information. [file 41598_2024_64088_MOESM1_ESM.pdf]

Supplementary data

C1s targeting antibodies inhibit the growth of cutaneous squamous carcinoma cells

Liisa Nissinen[1], Pilvi Riihilä[1], Kristina Viiklepp[1], Vaishnavi Rajagopal[2], Michael J. Storek[2], Veli-Matti Kähäri[1]

[1]Department of Dermatology and FICAN West Cancer Centre Research Laboratory, University of Turku and Turku University Hospital, Turku, Finland, Hämeentie 11 TE6, FI-20520 Turku, Finland

[2]Sanofi, Cambridge, MA, USA

Supplementary figures (S1-S6)

Supplementary Table S1

Original western blots for Figure 1., Figure 4., Supplementary Figure S4. and Supplementary Figure S5.

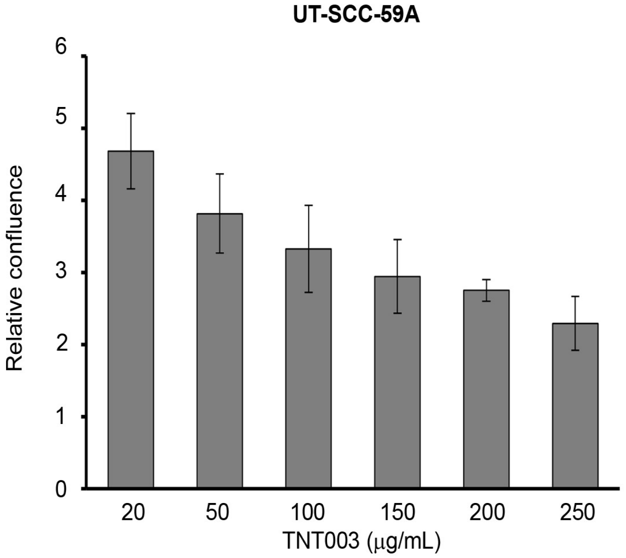

Supplementary Figure S1. The dose response effect of C1s targeted antibody TNT003 on the growth of cutaneous squamous cell carcinoma (cSCC) cells. cSCC cells (UT-SCC-59A) (7.5×10^3 cells/well) were plated on 96-well plates. Different concentrations of TNT003 were added to cells in serum free conditions. The IncuCyte S3 real-time cell imaging system was used to study the growth of cSCC cells and the relative confluence was analyzed by the instrument after 3 days (n=5-8).

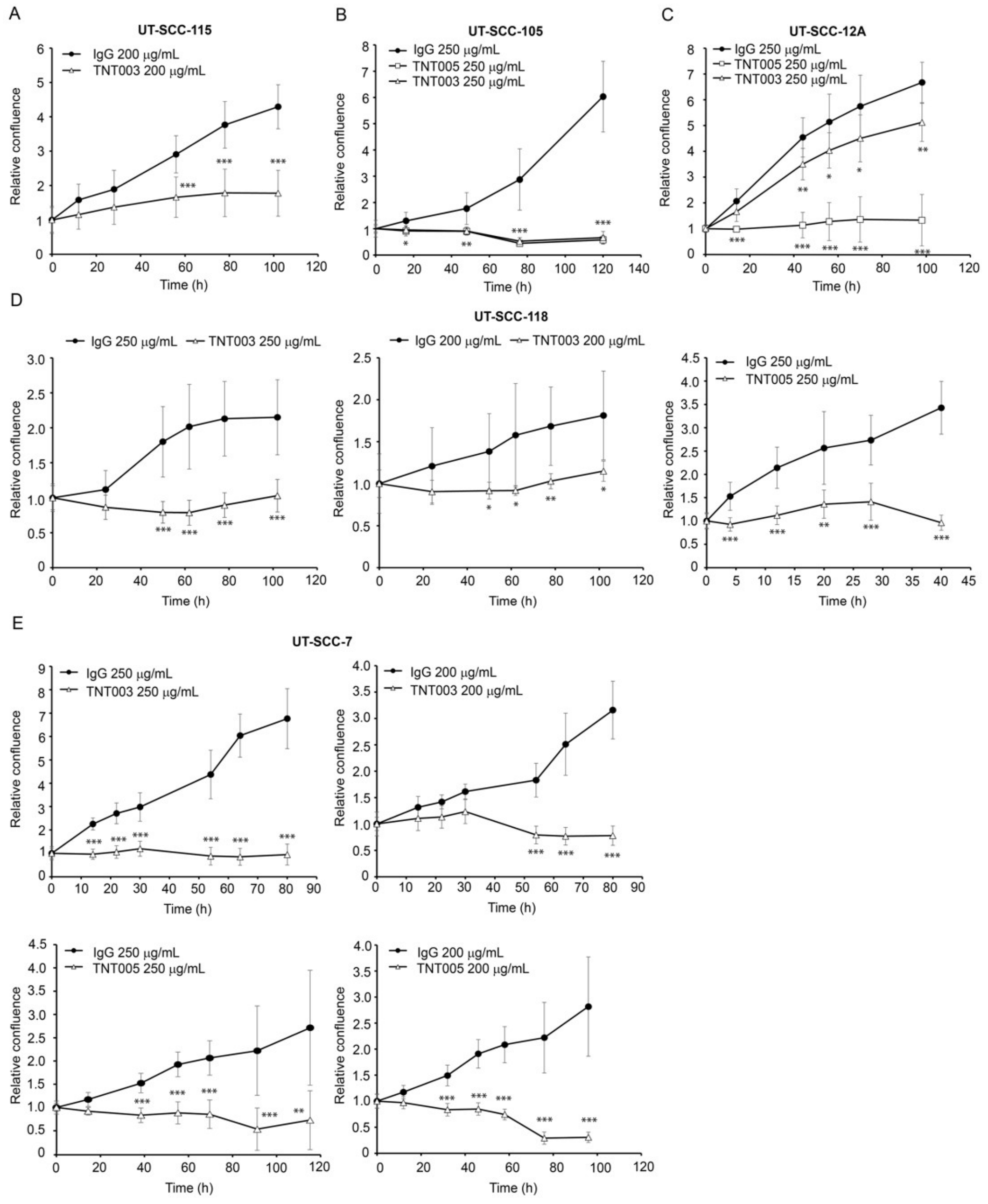

Supplementary Figure S2. C1s targeted antibodies TNT003 and TNT005 inhibit the growth of cutaneous squamous cell carcinoma (cSCC) cells. cSCC cell lines (**A**) UT-SCC-115, (**B**) UT-SCC-105, (**C**) UT-SCC-12A, (**D**) UT-SCC-118 and (**E**) UT-SCC-7 cells (7.5×10^3 cells/well) were plated on 96-well plates. Control antibody (IgG) and C1s targeting antibodies TNT003 and TNT005 (250 µg/mL or 200 µg/mL) were added to cells in serum free conditions. The IncuCyte S3 real-time cell imaging system was used to study the growth of cSCC cells and the relative confluence was analyzed by the instrument (n=5-8). $^{*}p < 0.05$, $^{**}p < 0.01$, $^{***}p < 0.001$, Student's t-test.

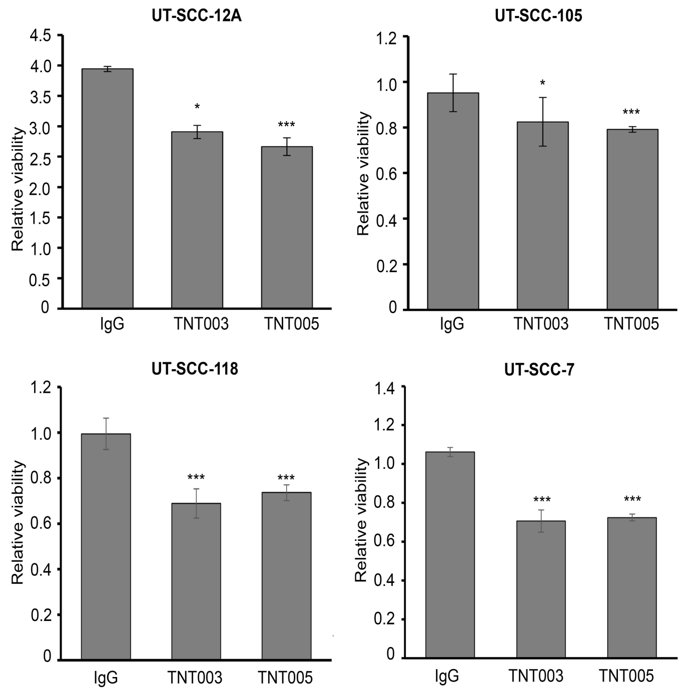

Supplementary Figure S3. C1s targeted antibodies TNT003 and TNT005 inhibit the viability of cutaneous squamous cell carcinoma (cSCC) cells. cSCC cells (1.0×10^4 cells/well) were plated on 96-well plates. Control antibody (IgG) and C1s targeting antibodies TNT003 and TNT005 were added to cells in serum free conditions (200 µg/mL). The number of cells was determined after 24h using CCK-8 assay (n = 6-8). $^{*}p < 0.05$, $^{***}p < 0.001$, Student's t-test.

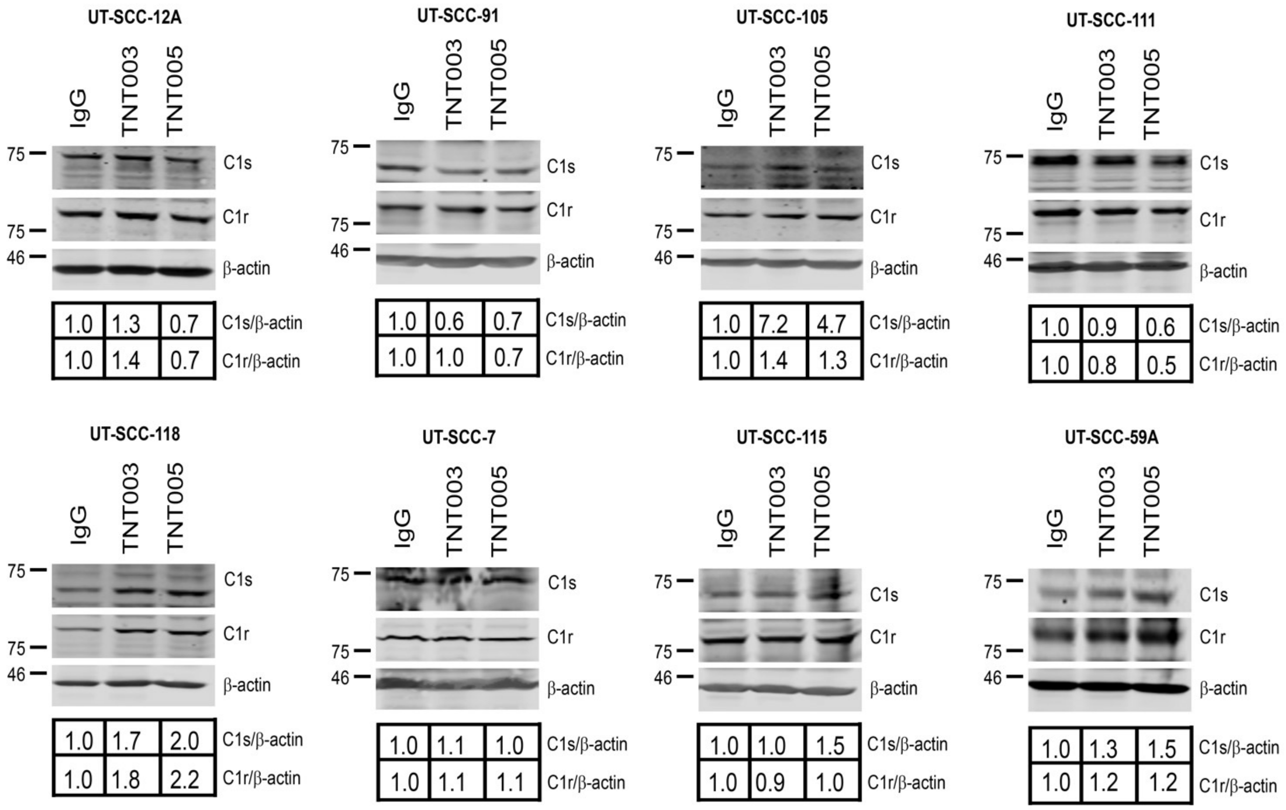

Supplementary Figure S4. Effect of C1s targeted antibodies TNT003 and TNT005 on C1s and C1r expression in cutaneous squamous cell carcinoma (cSCC) cells. cSCC cell lines were incubated with IgG, TNT003 or TNT005 (250 µg/mL) for 72 h. Total cell lysates were collected and analyzed by western blotting. The levels of C1s and C1r are shown. β-actin was determined as the loading control. Quantitations of the western blots corrected for loading controls are shown below the panels.

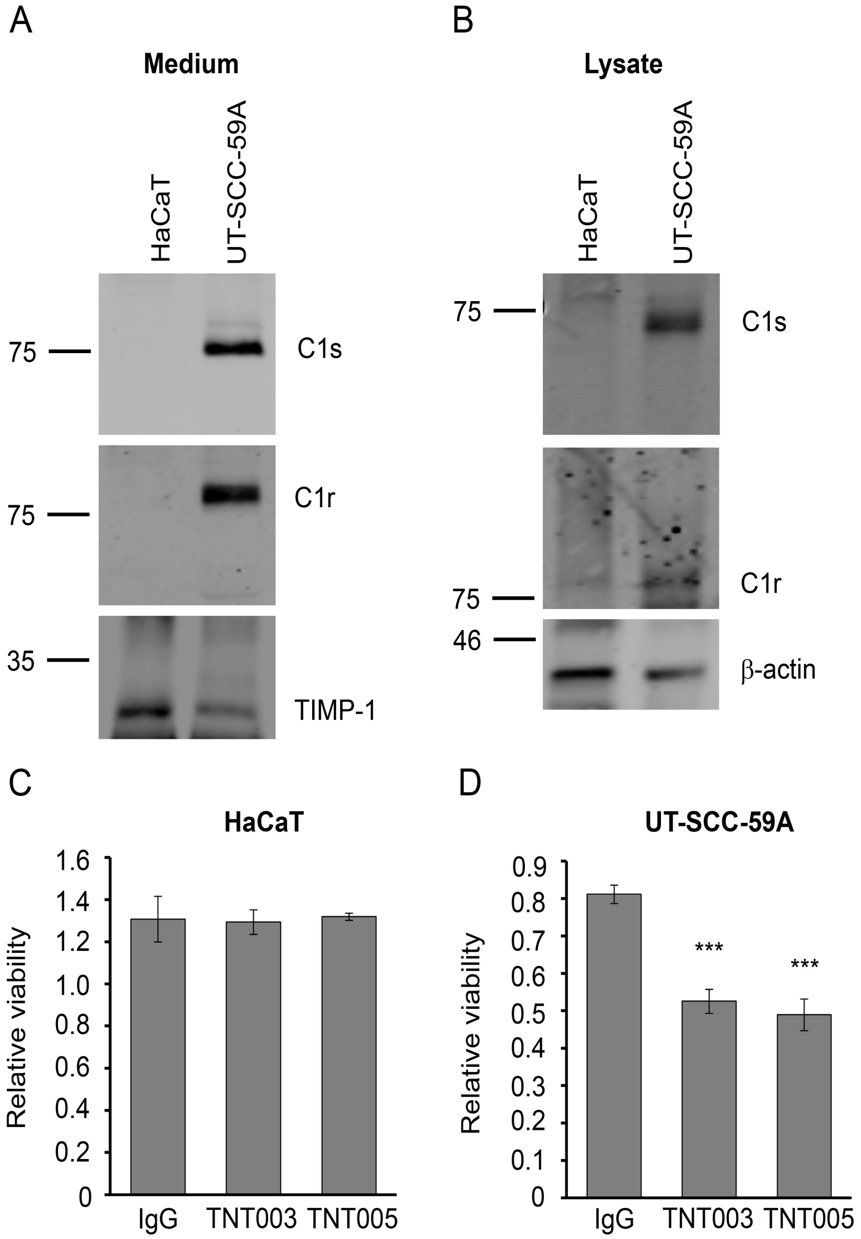

Supplementary Figure S5. C1s targeted antibodies TNT003 and TNT005 do not inhibit the viability of the immortalized non-tumorigenic human keratinocyte–derived cells (HaCaT). (A) Conditioned media and **(B)** total cell lysates of HaCaT and cSCC cells (UT-SCC-59A) were collected from cell growth experiments and analyzed by western blotting. The levels of C1s and C1r are shown. TIMP-1 **(A)** and β-actin **(B)** were determined as the loading controls. **(C)** HaCaT (7.5×10^3 cells/well) and **(D)** cSCC cells (UT-SCC-59A) (1.0×10^4 cells/well) were plated on 96-well plates. Control antibody (IgG) and C1s targeting antibodies TNT003 and TNT005 (250 μg/mL) were added to cells in serum free conditions. The viability of the cells was determined at 24h time point using CCK-8 assay (n = 6). $^{***}p < 0.001$, Student's t-test.

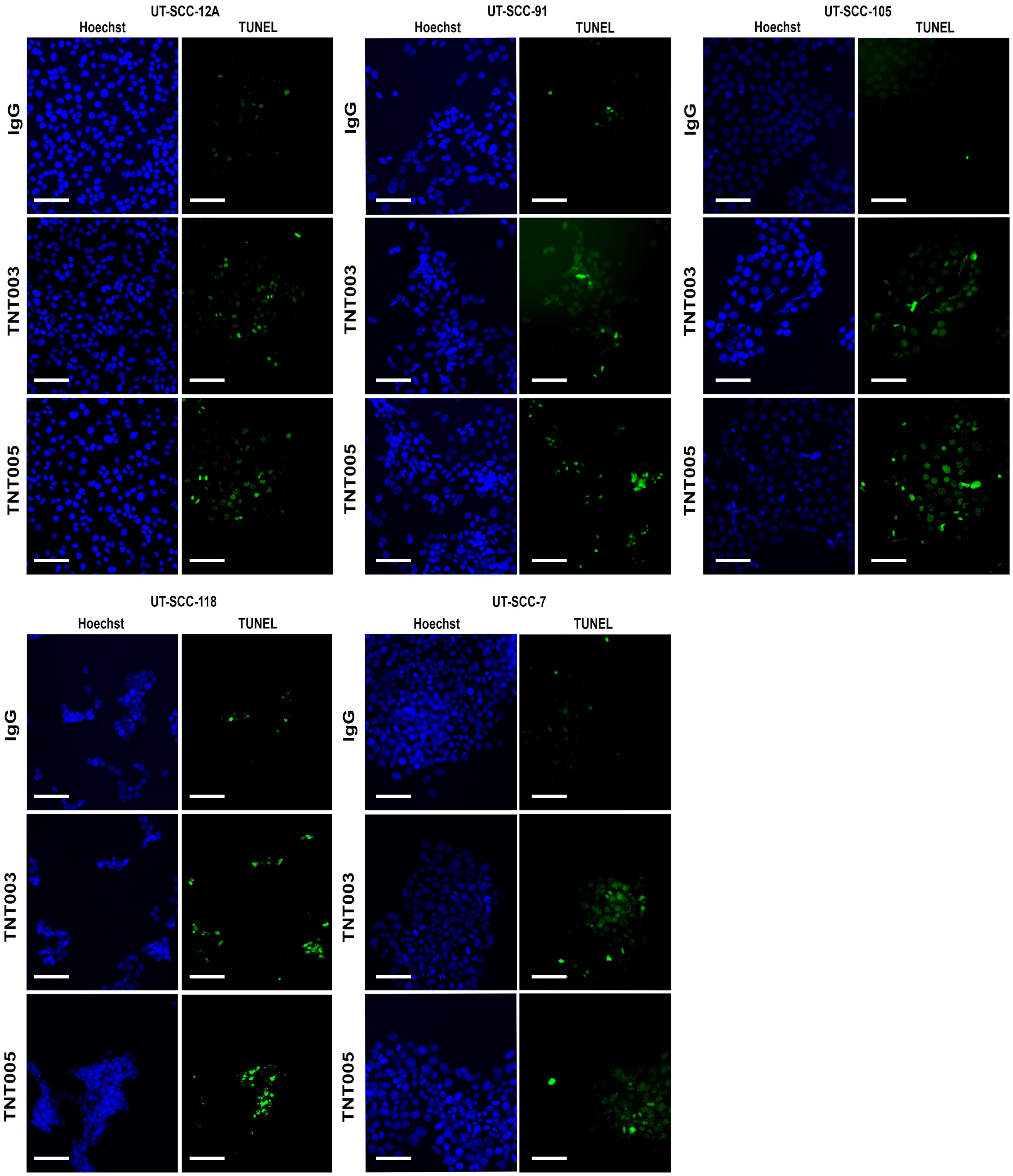

Supplementary Figure S6. C1s targeted antibodies TNT003 and TNT005 induce apoptosis of cutaneous squamous cell carcinoma (cSCC) cells. cSCC cell lines were treated with control antibody (IgG) and C1s targeting antibodies TNT003 and TNT005 (250 µg/mL) for 24 h under serum free conditions, apoptotic cells were detected with TUNEL staining. Representative images of Hoechst and TUNEL stainings are shown. Scale bar 100µm.

Table S1. Origin of cutaneous squamous cell carcinoma (cSCC) cell lines used in the study.

UT-SCC	Sex	Age	Location	Type of lesion	TNM	Grade
12A	Female	81	skin of nose	primary	T2N0M0	1
91A	Male	70	skin of nose	recurrent	T2N0M0	1
105	Female	84	face	primary	T1-T2N0M0	1-2
111	Male	89	face	primary	T3N0M0	3
118	Female	84	face	primary	T2N0M0	1
7	Male	68	temporal skin	metastasis/neck	T1N0M0	2
59A	Male	81	temporal skin	metastasis	rT1N3M0	3
115	Female	92	skin of ear	metastasis/neck	rT2N2M0	2

Original western blots

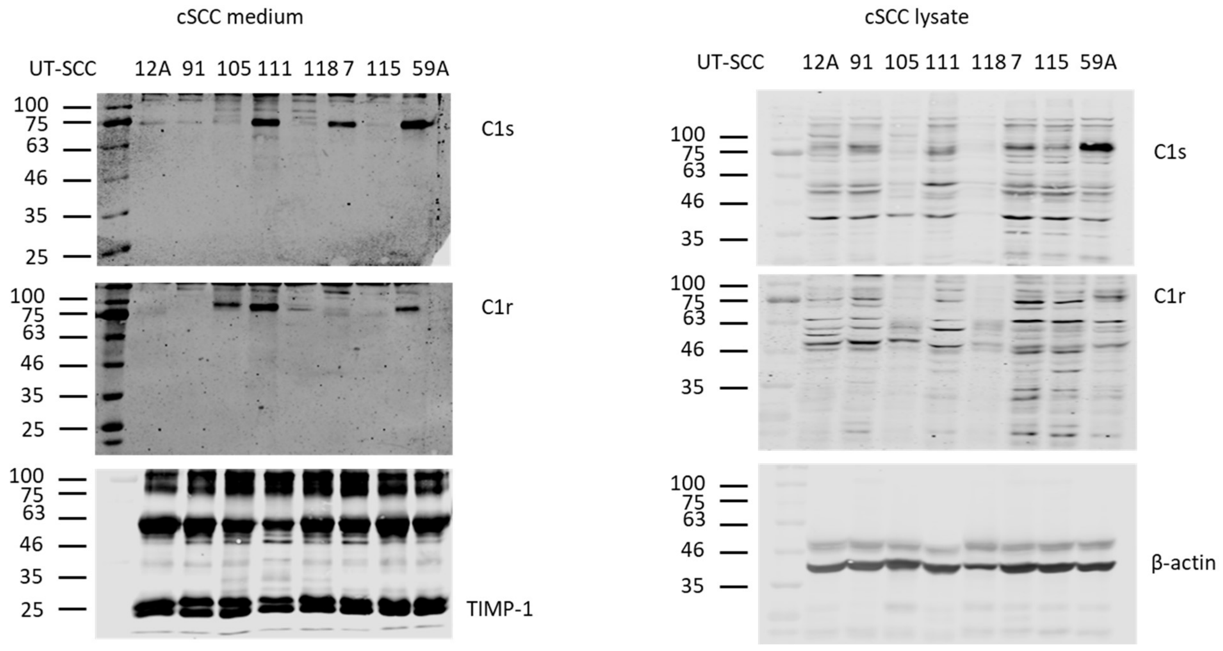

Figure 1.

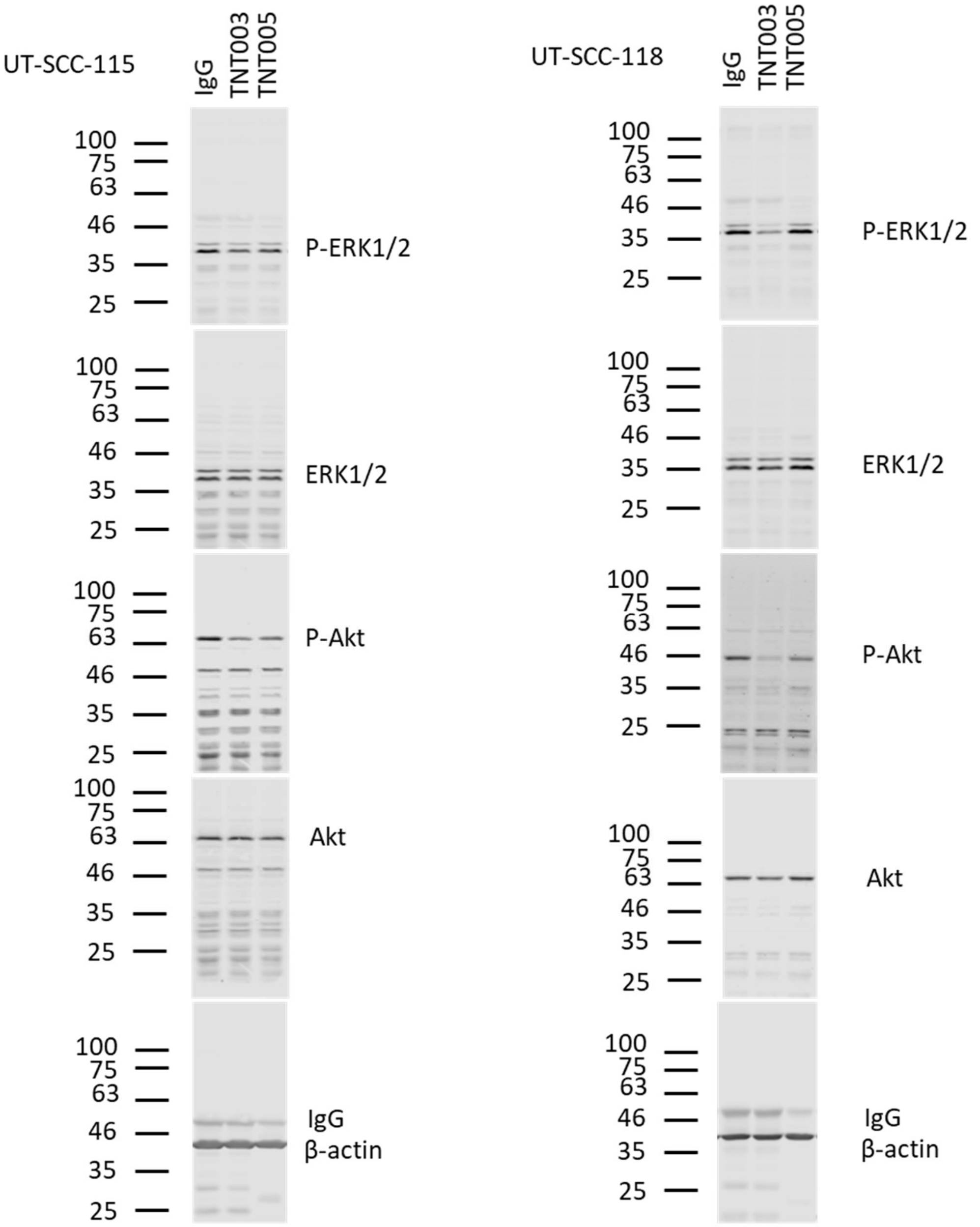
UT-SCC-115
IgG
TNT003
TNT005
UT-SCC-118
100
75
63
46
35
25
P-ERK1/2
ERK1/2
P-Akt
Akt
IgG
β-actin

Figure 4.

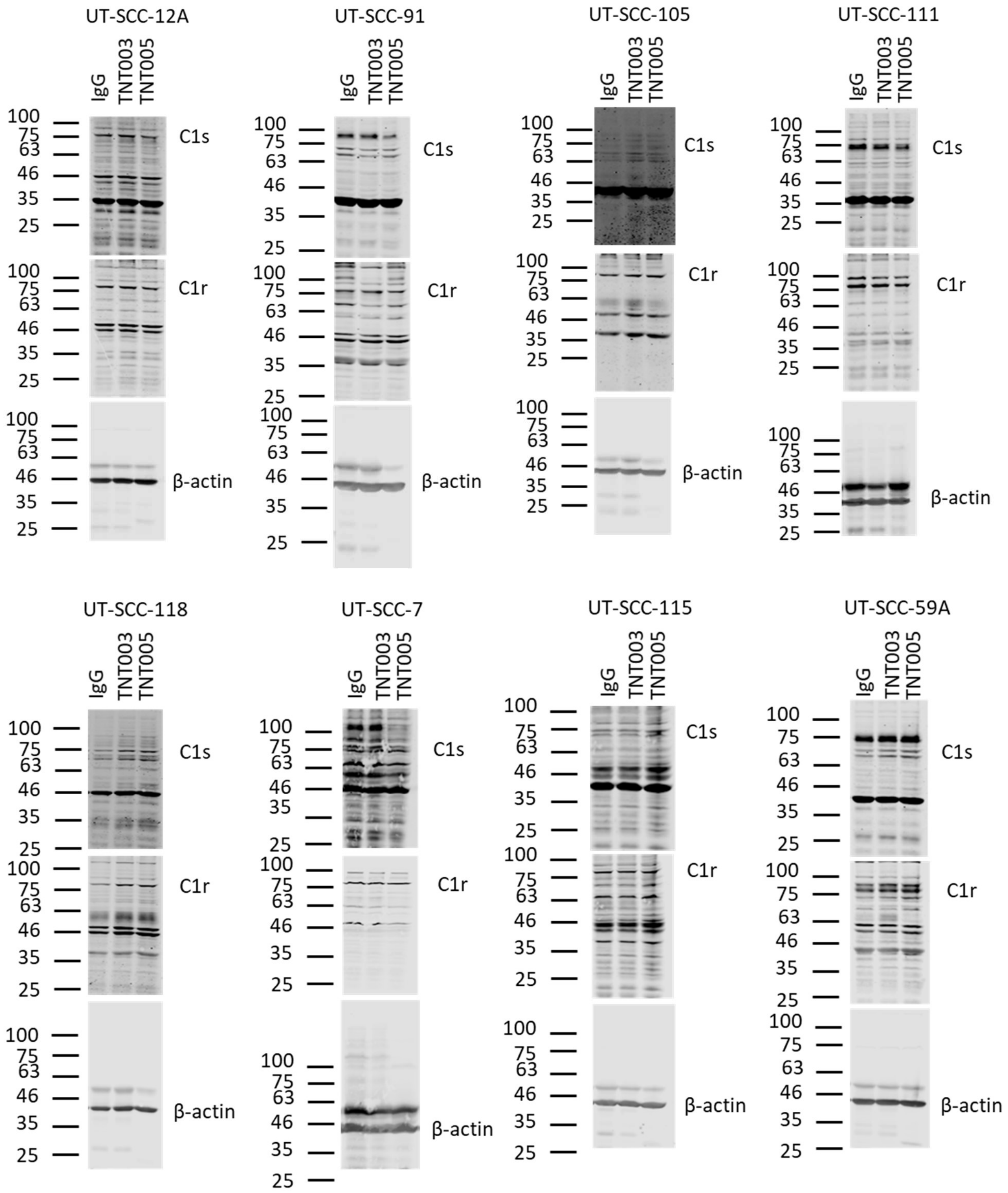

Supplementary Figure S4

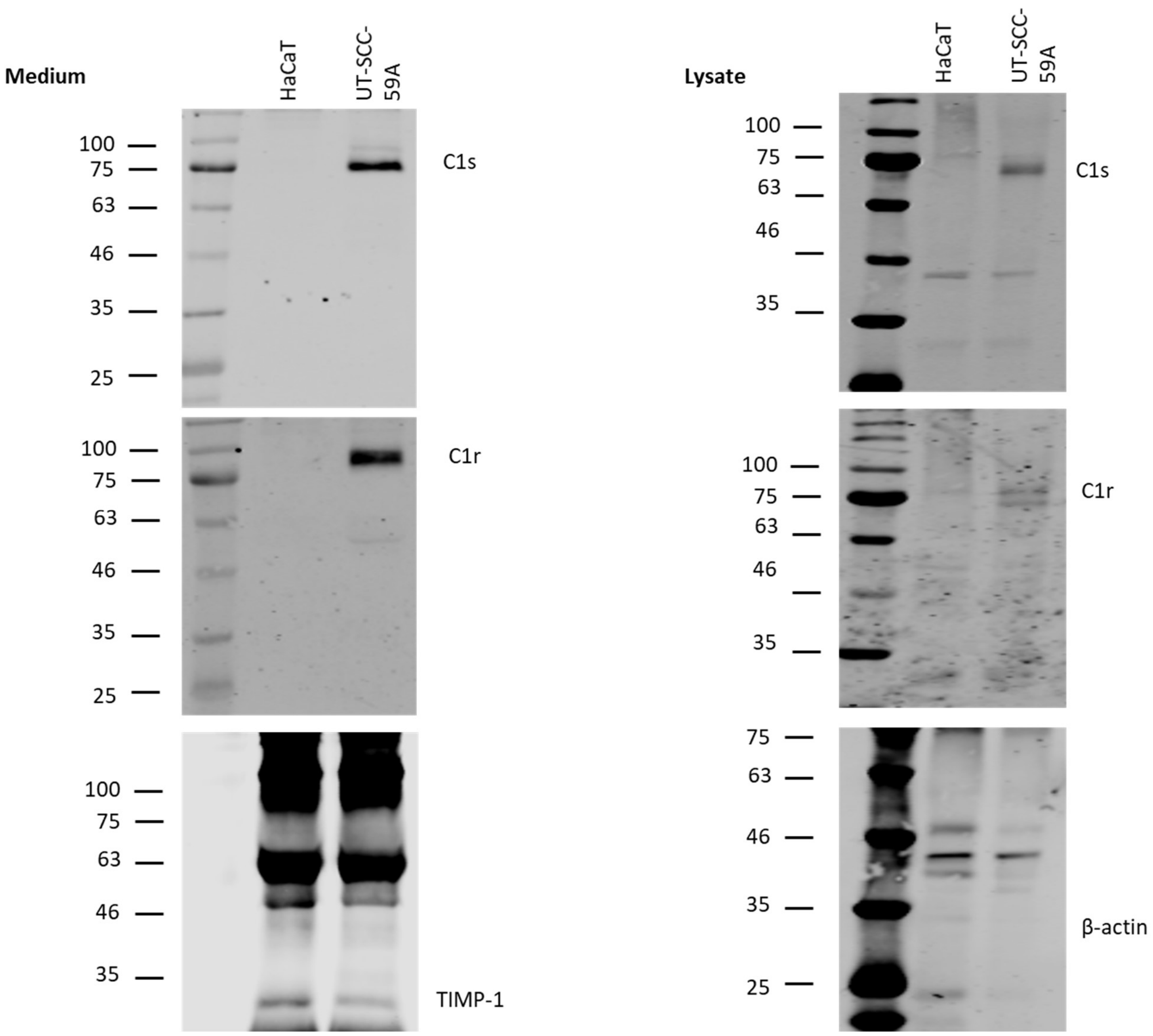

Supplementary Figure S5
